# Major Adverse Kidney Events Are Associated with the Aquaporin 5 -1364A/C Promoter Polymorphism in Sepsis: A Prospective Validation Study

**DOI:** 10.3390/cells9040904

**Published:** 2020-04-07

**Authors:** Lars Bergmann, Hartmuth Nowak, Winfried Siffert, Jürgen Peters, Michael Adamzik, Björn Koos, Tim Rahmel

**Affiliations:** 1 Klinik für Anästhesiologie, Intensivmedizin und Schmerztherapie, Universitätsklinikum Knappschaftskrankenhaus Bochum, In der Schornau 23-25, D-44892 Bochum, Germany; Lars.Bergmann@kk-bochum.de (L.B.); hartmuth.nowak@kk-bochum.de (H.N.); Michael.Adamzik@kk-bochum.de (M.A.); bjoern.koos@rub.de (B.K.); 2 Institut für Pharmakogenetik, Universitätsklinikum Essen, Hufelandstr. 55, D-45122 Essen, Germany; winfried.siffert@uk-essen.de; 3 Klinik für Anästhesiologie und Intensivmedizin, Universitätsklinikum Essen, Hufelandstr. 55, D-45122 Essen, Germany; juergen.peters@uni-duisburg-essen.de

**Keywords:** major adverse kidney events, acute kidney injury, sepsis, survival, aquaporin 5, *AQP5*, polymorphism

## Abstract

Since the functionally important *AQP5* -1364A/C single nucleotide promoter polymorphism alters key mechanisms of inflammation and survival in sepsis, it may affect the risk of an acute kidney injury. Accordingly, we tested the hypothesis in septic patients that this *AQP5* polymorphism is associated with major adverse kidney events and also validated its impact on 90-day survival. In this prospective observational monocentric genetic association study 282 septic patients were included and genotyped for the *AQP5* –1364A/C polymorphism (rs3759129). The primary endpoint was the development of major adverse kidney events within 30 days. In AC/CC genotypes, major adverse kidney events were less frequent (41.7%) than in AA genotypes (74.3%; OR:0.34; 95%-CI: 0.18–0.62; *p* < 0.001). Ninety-day survival was also associated with the *AQP5* polymorphism (*p* = 0.004), with 94/167 deaths (56.3%) in AA genotypes, but only 46/115 deaths (40.0%) in C-allele carriers. Multiple proportional hazard analysis revealed AC/CC genotypes to be at significantly lower risk for death within 90 days (HR: 0.60; 95%-CI: 0.42-0.86; *p* = 0.006). These findings confirm the important role of the *AQP5* -1364A/C polymorphism as an independent prognostic factor in sepsis. Furthermore, we demonstrate a strong association between this *AQP5* polymorphism and susceptibility for major adverse kidney events suggesting a promising characteristic in terms of precision medicine.

## 1. Introduction

Acute kidney injury (AKI) is a detrimental condition in critically ill patients and independently associated with morbidity and mortality [1,2]. AKI, characterized by a rapid decline in renal function, usually occurs as a complication of other severe medical conditions, such as sepsis [3]. In fact, AKI is a frequent sepsis-associated organ dysfunction [4,5], and a recent worldwide multicentric study with almost 8000 critically ill patients reported an AKI incidence of more than 60% within the first 72 h after sepsis onset [6]. Furthermore, duration and severity of AKI in sepsis are recognized as important risk factors for adverse outcomes despite various efficient renal replacement therapies (RRT). Accordingly, AKI and its sequelae still remain a challenge [7,8], and patients are stratified for distinct AKI phenotypes associated with either recovery or poor prognosis [1,8]. 

However, although several clinical risk factors for the development of AKI have been identified, the pathogenesis and mechanisms of AKI itself remain poorly understood [9]. Genetic factors have been proposed to contribute to interindividual differences of susceptibility to and recovery from AKI relevantly [10]. A promising candidate gene for investigation of sepsis-associated AKI is the water channel Aquaporin 5 (AQP5) since AQP5 is not only associated with transcellular and renal fluid transport [11], but also with cell proliferation [12] and key mechanisms of inflammation [13,14]. In humans, an altered AQP5 expression is linked to a common single nucleotide polymorphism (SNP; -1364A/C; rs3759129) in the AQP5 gene promoter. The substitution of cytosine for adenosine in the heterozygous AC and homozygous CC mutants was associated with decreased AQP5 expression compared with the AA wildtype [15]. Our previous studies already elucidated that the AQP5 genotype is associated with outcome in sepsis and acute respiratory distress syndrome (ARDS), both suggesting a protective role of the C-allele [16,17,18]. C-allele carriers, i.e., the heterozygous AC and homozygous CC mutants, were associated with a better 30-day survival in sepsis and in ARDS compared with AA genotypes [16,17]. Furthermore, we recently demonstrated an association between the AQP5 -1364A/C SNP and susceptibility for acute kidney injury in patients suffering from ARDS [18]. Thus, the AQP5 -1364A/C promoter SNP may also impact on the development of AKI related outcomes in sepsis that can compositely be assessed as major adverse kidney events within 30 days (MAKE30) [19]. However, data validating the association with survival in an independent cohort of septic patients and demonstrating an impact on AKI related outcomes are lacking. 

Accordingly, we hypothesized that AC/CC genotypes of the AQP5 -1364A/C promoter polymorphism are associated with a lower risk for MAKE30 (i.e., incidence for AKI, death, new RRT, and persistent renal dysfunction within the first 30 days) and also demonstrate higher 90-day survival. 

## 2. Materials and Methods

### 2.1. Study Design and Oversight

The study was reviewed and approved by the Ethics Committee of the Medical Faculty of the University of Duisburg-Essen (protocol no. #06-3078) and written informed consent was obtained from patients or their guardians, as appropriate, and conducted in accordance with the Declaration of Helsinki, good clinical practice guidelines, and local regulatory requirements. In this prospective, monocentric, observational study, we enrolled adult septic patients treated in the intensive care unit (ICU) of the University of Duisburg-Essen between October 1, 2009, and September 30, 2014. Patients were then stratified according to their genotypes of the AQP5 -1364A/C single nucleotide promoter polymorphism. 

### 2.2. Patient Population and Treatments

Patients were considered eligible if they fulfilled the criteria for severe sepsis as defined by Bone et al., and enrolment was completed within the first 24 h after criteria for severe sepsis were met [20]. In addition, all included patients also met the criteria of the current SEPSIS-3 definition [21]. Exclusion criteria were age under 18 years, pregnancy, pre-existing severe chronic kidney disease (Kidney Disease Improving Global Outcomes (KDIGO) category ≥ G4), RRT before enrollment, and decision to withhold or withdraw life-sustaining therapies on the day of study inclusion. Briefly, two hundred eighty-two patients suffering from sepsis (174 males, 108 females) were included in this study during the 4-year recruitment period. Patients were treated using analgesia and sedation, fluid administration and lung-protective mechanical ventilation, anticoagulation, as well as hemodynamic, antibiotic, and diagnostic management, as needed and as described previously [16]. 

### 2.3. Data Collection

Clinical and demographic data were mainly collected during routine care and extracted from hospital and ICU health records. These data were transferred pseudonymized to the study’s specific database, which included information on demographic characteristics, pre-existing morbidities, Simplified Acute Physiology Score II (SAPS-II), Sepsis-Related Organ Failure Assessment Score (SOFA), medications of vasoactive drugs, blood chemistry values, net fluid balance, degree of AKI according the KDIGO classification [22], necessity for RRT, and vital status on hospital discharge and day 90. Baseline characteristics were collected during the first 24 h after the diagnosis of sepsis was made (Table 1). All patients were observed for 30 days regarding MAKE30 and additionally for 90-day survival, both calculated from ICU admission with the diagnosis of sepsis. 

### 2.4. DNA-Genotyping

DNA was extracted from whole blood using the QIAamp-Kit (QIAGEN, Hilden, Germany). For genotyping the SNP -1364A/C in the AQP5 promoter, a polymerase chain reaction was performed with the forward AQP5-SE 5´-GAAACTGCAGGATGAGAGAAAT-3´ and the biotinylated reverse AQP5-AS 5´-TCTCTGTTCTCCACCTCTCCA-3´, followed by pyrosequencing, as described previously [15,16]. 

### 2.5. Study Groups and Endpoints

All sepsis patients were allocated depending on the AQP5 -1364A/C promoter polymorphism to the AA genotype (wildtype) or the AC/CC genotype (mutants). Homozygous and heterozygous C allele carriers were analyzed together in terms of our a priori defined study endpoints due to the very low frequency of the CC genotype in appreciation of our previous studies [16,17].

The primary endpoint was the proportion of patients who met one or more criteria for a MAKE30 [19,23]—the composite of death, new receipt of RRT, or persistent renal dysfunction (defined as a final inpatient creatinine concentrations ≥200% of its baseline). Baseline creatinine concentration was estimated with a previously described three-variable formula [24]. Secondary renal and clinical outcomes were followed for day 30 or censored by death or ICU-discharge whichever came first. Secondary renal outcomes included AKI as defined by the KDIGO criteria [3], RRT free days, the highest creatinine serum concentration during ICU stay, and the final creatinine concentration. Secondary clinical outcomes included ICU-free days, as well as hospital length of stay, ventilator-free days, and the net fluid balance. In addition, survival was followed for 90-days after sepsis onset. 

### 2.6. Statistical Analysis

The characteristics of the patients at baseline were reported as percentages for categorical variables and as means with standard deviations (±SD) or medians with interquartile ranges (25th; 75th percentile) for continuous variables, as appropriate. Categorical variables were compared using Chi-square or Fisher’s exact test, and continuous variables by using Student’s *t*-tests or non-parametric Wilcoxon–Mann–Whitney-tests. All analyses were conducted with a two-sided alpha error *p* of 0.05, and all confidence intervals (CI) were calculated with coverage of 95%.

The potential association between the AQP5 -1364A/C promoter SNP genotypes and the primary and secondary outcomes was assessed using multiple logistic regression analysis. The regression model was adjusted for age, sex, chronic kidney disease, SOFA-score, procalcitonin concentration, and septic shock as pertinent confounders showing a significant difference in the univariate analysis (Supplementary Materials Table S1). 

In addition, the clinical endpoint of 90-day survival was graphically assessed by the Kaplan–Meier method. The log-rank test was used to evaluate the univariate relationship between the AQP5 SNP and 90-day survival. Afterward, a multivariate Cox regression analysis was used to assess the joint impact of the AQP5 genotype as well as patients’ sex, age, chronic kidney disease, vasopressor support, mechanical ventilation, net fluid balance, serum lactate concentration, total bilirubin concentration, AKI, and procalcitonin concentration as potential predictors for 90-day survival (Supplementary Materials Table S2). At first, a Cox regression was performed with several models with a single predictor. Thereafter, an initial multivariate model investigated all main effects simultaneously. To avoid overfitting, a restricted model with only five variables was assessed afterward using only predictors with a *p*-value of ≤0.05 in the univariate and the initial multivariate model.

All analyses were performed using SPSS (version 25, IBM, Chicago, IL, USA). For graphical presentations, GraphPad Prism 8 (Graph-Pad, San Diego, CA, USA) was used.

## 3. Results

A total of 282 septic patients were enrolled. The mean age was 56.9 years (±15.3), and 61.7 % (174/282) patients were male. At the time of study inclusion, more than 80% (228/282) received mechanical ventilation, and almost 90% received vasopressors. The mean SOFA-score at baseline was 12.1 (±4.4), and more than 21.6% (61/282) of the patients already fulfilled the Sepsis-3 criteria for septic shock. 

Regarding the distribution of the genetic variation of the AQP5 SNPs according to the Hardy–Weinberg equilibrium, we observed 167 AA genotypes (59.2%), 100 for the AC genotype (35.5%), and 15 for the CC genotype (5.3%). Accordingly, there was no deviation from the Hardy–Weinberg equilibrium (*p* = 0.77). 

Upon ICU admission, AA genotypes (52.1%; 87/167) and AC/CC genotypes (49.6%; 57/115) showed comparable frequencies of an AKI-score ≥ 1 (*p* = 0.676; Table 1). In addition, no differences were found between AA and AC/CC genotypes regarding baseline serum creatinine concentration (*p* = 0.734), mechanical ventilation (*p* = 0.22), vasopressor support (*p* = 0.228), or SOFA score (*p* = 0.716; Table 1). Moreover, there were no statistically significant differences in other baseline characteristics between AA and AC/CC AQP5 promoter SNP genotypes (Table 1).

**Figure 1:**
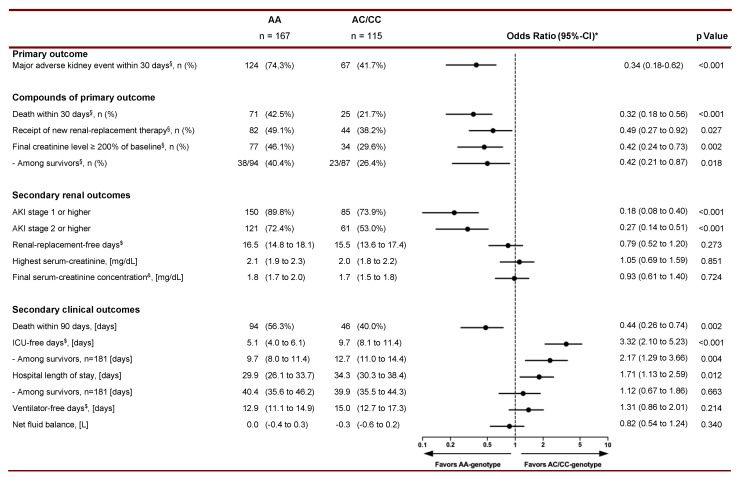
Analysis of rates and odds ratios for primary, secondary renal, and secondary clinical outcomes in septic patients stratified for their AQP5 -1364 A/C promoter polymorphism. Data are presented as n (%) or mean (with 95%-CI). AKI: Acute kidney injury; ICU: Intensive care unit. The odds ratio was calculated with AA serving as the reference group. Therefore, odds ratios of more than 1.0 indicate a higher frequency in AC/CC genotypes, an odds ratio of less than 1 indicate a higher frequency in AA genotypes. * Odds ratios were adjusted for pertinent confounders (i.e., age, sex, chronic kidney disease of stage 3 or higher, Sepsis-Related Organ Failure Assessment (SOFA) score, procalcitonin-concentration, and septic shock). ^§^ A major adverse kidney event is defined as a composite endpoint of death, receipt of new renal-replacement therapy, or final creatinine level that was at least 2-fold higher than the estimated baseline level within 30 days. ^$^ Renal-replacement-free days, ICU-free, and ventilator-free days refer to the number of days on which the patient was alive and free from the specific therapy within the first 30 days after enrollment. ^&^ Final creatinine concentration on day 30, ICU-discharge, or before death, whichever occurred first.

Despite this homogeneity at baseline, 124 of 167 AA genotypes (74.3%) but only 67 of 115 C-allele carriers (41.7%) had a MAKE30 within 30 days after sepsis onset (odds ratio: 0.34; 95%-CI: 0.18 to 0.62; *p* < 0.001; Figure 1). In detail, all three components of the composite endpoint MAKE30 were less frequent in AC/CC compared to AA genotypes (Figure 1), namely death (odds ratio: 0.32; 95%-CI: 0.18 to 0.56; *p* < 0.001), receipt of new RRT (odds ratio: 0.49; 95%-CI: 0.27 to 0.92; *p* = 0.027), and persistent renal dysfunction (odds ratio: 0.42; 95%-CI: 0.24 to 0.73; *p* = 0.002).

Furthermore, 73.9% (85/115) of AC/CC genotypes had a distinctly lower incidence of AKI stage 1 or higher compared to 89.8% (150/167) of AA genotypes (odds ratio: 0.18; 95%-CI: 0.08 to 0.40; *p* < 0.001; Figure 1). This was accompanied by more ICU-free days in C-allele carriers compared to the AA genotypes (odds ratio: 3.32; 95%-CI: 2.10 to 5.23; *p* < 0.001; Figure 1). 

**Figure 2:**
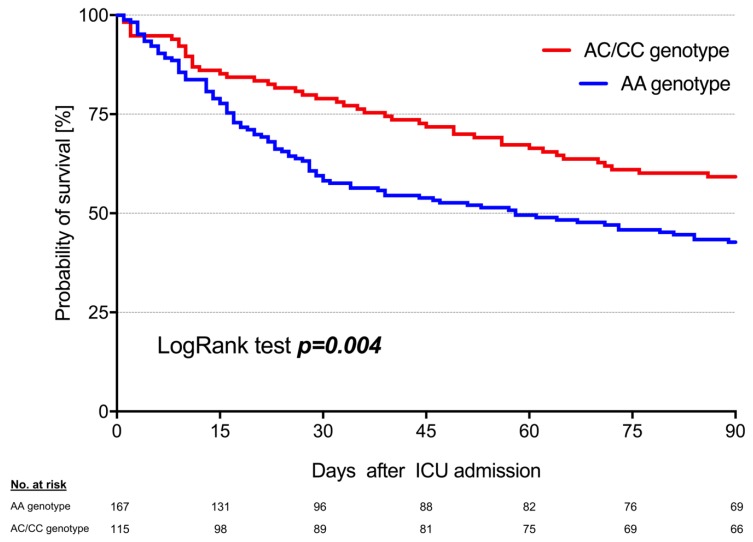
Ninety-day survival in patients with sepsis stratified for their AQP5 -1364 A/C promoter polymorphism. Kaplan–Meier estimates were used to calculate probabilities of 90-day survival based on Aquaporin 5 SNP. Ninety-day survival was higher in C-allele carriers compared with AA genotypes. ICU, intensive care unit.

Moreover, 90-day survival was higher in AC/CC genotypes (56.3%; 94/167) compared to AA genotypes (40.0%; 46/115) of the AQP5 -1364A/C SNP (Figure 2, *p* = 0.004). In addition, multivariate Cox regression analyses revealed the AQP5 genotype status to be an independent and strong prognostic factor when jointly considering with other predictors of 90-day survival (Table 2). In this context, C-allele carriers had a 40% decreased risk of death within 90 days compared to AA genotypes (hazard ratio: 0.60; 95%-CI: 0.42 to 0.86; *p* = 0.006). The cumulative net fluid balance was also associated with the patient’s outcome (Table 2). Patients with a net fluid balance of less than -1 L had a more than 2-fold lower risk of death within 90 days (hazard ratio: 0.42; 95%-CI: 0.26 to 0.68; *p* < 0.001) than patients with a net fluid balance of more than +1 L who had a nearly 2-fold greater risk (hazard ratio: 1.74; 95%-CI: 1.20 to 2.53; *p* = 0.004) in comparison to those with a neutral net fluid balance (−1 L to +1 L). 

## 4. Discussion

The major finding of this study is that the AA genotype of the AQP5 -1364A/C SNP is associated with an approximately 3-fold greater risk for MAKE30 in septic patients. This is accompanied by a 5-fold higher incidence of AKI and increased 90-day mortality in AA genotypes. Since the AQP5 -1364A/C promoter SNP was previously described as an independent prognostic factor in sepsis [16]. We now can confirm and extend our prior findings by demonstrating an approximately 1.7-fold greater risk of death within 90-days in this new and independent cohort of septic patients. 

While recent research has enhanced our knowledge of the pathobiology of sepsis-associated AKI [25], the AKI morbidity and mortality in sepsis remains unacceptably high, and studies illuminating further risk factors are needed to allow for better stratification of preventive strategies [7]. In this study, we considered an altered AQP5 expression documented to be evoked by the common AQP5 -1364A/C promoter SNP as a promising risk factor for sepsis-associated AKI. This AQP5 SNP has already been described as an important prognostic factor regarding 30-day survival in septic patients [16]. Moreover, the C allele of the AQP5 SNP independently showed a lesser risk for and also a better resolution of AKI in patients with bacterial evoked ARDS [18]. The present study, covering a broad spectrum of septic patients, revealed a significantly lower AKI incidence, fewer major adverse kidney events within 30 days after onset of sepsis, and a higher recovery rate of kidney function in AC/CC genotypes. Therefore, the AQP5 -1364 A/C SNP presumably contributes to the development and recovery of AKI that in turn may impact both mortality and morbidity in septic patients. 

Although the exact mechanisms linking the AQP5 -1364A/C SNP to AKI and outcome in sepsis cannot be pinpointed by this observational study, a few speculations can be made. AQP5 is expressed in type B intercalated cells of renal collecting ducts and, thereby, may contribute to the maintenance of a normal urine osmolarity and support excretory function [26]. In this context, investigators also reported an association of the net fluid balance with AKI and even outcome in sepsis [27,28]. The latter is consistent with our data since we found that a negative fluid balance (<−1 L) was associated with a lesser 90-days mortality and, in turn, a positive fluid balance (>+1 L) was associated with a greater 90-days mortality. However, the AQP5 promoter polymorphism itself did not statistically impact on cumulative net fluid balance. Thus, the impact on MAKE, as well as the incidence and resolution of an impaired renal function in sepsis, are presumably unrelated to APQ5 effects on renal excretory function and fluid balance. This assumption is supported by recent studies of renal AQP5 expression in situ in mice, rats, and humans that failed to reveal an impact of AQP5 for active transepithelial fluid absorption under normal conditions [26] or in acute lung injury [29]. 

Current research has addressed the involvement of the renin–angiotensin–aldosterone system (RAAS) in sepsis and AKI. Growing evidence suggests that locally produced intrarenal angiotensin II, a key effector peptide of RAAS, might contribute to AKI by increasing the expression of pro-inflammatory and pro-fibrotic cytokines and thereby modulating local inflammation [30,31]. In this context, we previously described that the AQP5 -1364A/C SNP alters the regulation of the RAAS in young, healthy humans as well as in patients with coronary heart disease [15]. The C-allele was associated with greater RAAS suppression, and this RAAS downregulation was blunted in AA genotypes [15]. Thus, the association of the AQP5 SNP with altered survival and AKI incidence in sepsis could be the result of a different RAAS suppression among the AQP5 genotypes. However, we also previously showed that the AQP5 -1364A/C polymorphism is not associated with altered plasma angiotensin or serum aldosterone concentrations in adults experiencing sepsis [16]. Thus, the impact of the AQP5 expression on AKI seems not to be mediated by an altered RAAS system but other causes. 

Although the exact immunologic mechanisms remain incompletely understood, it is also evident that sepsis increases the expression of inflammatory cytokines and alters leukocyte reactivity [32]. This, in turn, may orchestrate deleterious effects on the kidney, e.g., by renal capillary plugging and micro-thrombi [33]. Certainly, AQP5 significantly impacts upon key mechanisms of inflammation that prevail in sepsis, including immune cell migration and proliferation [13,14]. AQP5 expression facilitates cell movement by transient formation of membrane protrusions via water entry (lamellipodia and membrane ruffles) [14], and target-oriented human neutrophil migration is faster and more frequent with increased AQP5 expression in the AA genotype of the AQP5 -1364A/C SNP [13]. Since C-allele carriers are associated with a lower AQP5 expression in sepsis compared to the AA genotypes [16], the AC/CC genotypes may confer an altered immune response. This C-allele shaped host response may thus be characterized by an attenuated inflammatory chain and decelerated immune cell migration evoking less collateral damage on the kidneys under septic conditions. 

Accordingly, we assume that increased mortality in AA genotypes in sepsis is more likely to be mediated by aggravated inflammation or a more deleterious host response than due to the role of AQP5 in renal water transport, fluid balance, or impact on the RAAS system. Thus, the mechanistic causality of the described association in the present study needs to be elucidated at the basic research level in upcoming studies. Nevertheless, with this study, we can confirm the impact of the AQP5 -1364A/C SNP as a strong and independent prognostic marker that seems to be strongly associated with renal associated outcomes. Thus, a genotype-based adjustment of diagnostics and treatments in terms of precision medicine in the near future seems prudent.

Our study has limitations that should be mentioned. First, the observational design and lack of histologic and mechanistic examinations preclude verification about causality and mechanisms. Second, the study was conducted at a single academic center, which may limit its generalizability. However, the single-center nature of this study might also be an advantage as it limits protocol variability, in particular, when treating highly complex diseases, such as sepsis. Third, despite progress in AKI definition by KDIGO criteria [34], the sensitivity and accuracy of AKI criteria have not been generally acknowledged. Thus this inherent limitation also applies to our study. Fourth, no formally accepted definition of renal recovery and AKI-related outcome exists; thus, a clinically meaningful endpoint is elusive [35]. However, we selected the composite endpoint MAKE30 to solidify the importance of AKI-related recovery and outcome, as previously recommended [19]. Fifth, our study with few exceptions was conducted in patients of European–Caucasian descent; thus, findings cannot be simply generalized to subjects of other ancestries. 

## 5. Conclusions

In septic patients, the AC/CC genotype of the functionally relevant AQP5 -1364A/C polymorphism was associated with a favorable effect on the composite outcome of death, newly installed renal replacement therapy, and persistent renal dysfunction. Furthermore, we can confirm and extend our prior findings by demonstrating an approximately 1.7-fold greater risk of death within 90 days in this new and independent cohort of septic patients. Consequently, this study highlights the water channel AQP5 and its related gene polymorphism as a promising diagnostic and therapeutic target in the age of precision medicine.

## Figures and Tables

**Table 1:** Baseline characteristics of septic patients at baseline and stratified by AQP5 -1364A/C genotype (n = 282).

Characteristic	AA		AC/CC		
	(n = 167)		(n = 115)		*p*-Value
Age [years]	56.4	(±15.5)	57.6	(±16.4)	0.521
Sex, male [n]	103	(56.8%)	71	(59.6%)	0.958
Body mass index [kg/m2]	27.2	(±6.0)	26.9	(±5.2)	0.616
Ethnicity [n]					0.830
- Caucasian	157	(94.0%)	112	(97.4%)	
- Other	10	(6%)	3	(2.6%)	
Medical history [n]					
- Cardiovascular disease	89	(53.3%)	67	(58.3%)	0.410
- Pulmonary disease	46	(27.5%)	25	(21.7%)	0.269
- Diabetes mellitus	31	(18.6%)	20	(17.4%)	0.936
- Gastrointestinal disease	23	(13.8%)	18	(15.7%)	0.617
- History of malignant disease	14	(8.4%)	11	(9.6%)	0.612
Renal conditions					
- CKD of stage 3 or higher^$^ [n]	22	(13.2%)	12	(10.4%)	0.487
SAPS II score	41.1	(±17.8)	42.7	(±18.7)	0.471
SOFA score	12.1	(±4.4)	12.0	(±4.5)	0.958
Septic Shock [n]	32	(19.2%)	29	(25.2%)	0.225
AKI stage 1 or higher	87	(52.1%)	57	(49.6%)	0.228
Vasopressor support [n]	149	(89.2%)	97	(84.3%)	0.228
Mechanical ventilation [n]	139	(83.2%)	89	(77.4%)	0.220
Net fluid balance^§^ [L]	0.0	(−1.5 to 1.5)	−0.3	(−1.9 to 1.3)	0.409
Procalcitonin concentration [pg/mL]	4.4	[1.7–12.7]	3.1	[1.2–17.1]	0.491
C-reactive protein concentration [mg/dL]	13.8	[7.4–20.7]	13.6	[7.4–23.6]	0.791
Leukocyte concentration [nL^-1^]	13.3	[9.0–20.2]	13.4	[8.7–19.6]	0.888
Creatinine concentration [mg/mL]	1.36	[0.82–2.12]	1.33	[0.88–1.99]	0.734
Blood urea nitrogen [mg/dL]	19.6	[11.7–28.5]	18.7	[12.6–25.2]	0.796
Hemoglobin [g/dL]	9.4	[8.7–10.5]	9.6	[8.9–10.6]	0.284
Total bilirubin concentration [mg/dL]	1.1	[0.4–2.5]	1.0	[0.4–2.2]	0.715
Serum lactate concentration [mg/dL]	1.3	[0.8–1.8]	1.4	[0.6–2.0]	0.577
Etiology of infection [n]					
- Pneumonia	63	(37.7%)	43	(37.4%)	0.999
- Urinary tract infection	41	(24.5%)	30	(26.1%)	
- Abdominal infection	26	(15.6%)	18	(15.7%)	
- Skin or muscle infection	9	(5.4%)	5	(4.3%)	
- Bloodstream infection	7	(4.2%)	5	(4.3%)	
- Other / unknown origin	21	(12.6%)	14	(12.2%)	
Blood cultures [n]					0.919
- Gram-positive isolates	46	(27.5%)	33	(28.7%)	
- Gram-negative isolates	48	(28.7%)	28	(24.3%)	
- Fungal isolates	6	(3.6%)	5	(4.3%)	
- Mixed isolates	34	(20.4%)	27	(23.5%)	
- Negative blood cultures	33	(19.8%)	22	(19.2%)	

The data are presented as n (%), mean (± SD), or median (25th-75th percentile). CKD: Chronic kidney disease; AKI: Acute kidney injury; SOFA score: Sepsis-Related Organ Failure Assessment score; SAPS II score; Simplified Acute Physiology score. The following missing data were excluded from the analysis: 4 case missing for body mass index; 5 cases were missing for SAPS II score; 8 cases were missing for procalcitonin concentration; 16 cases missing for C-reactive protein concentration; 7 cases missing for leukocyte concentration; 17 cases missing for blood urea nitrogen. ^$^ Chronic kidney disease of stage 3 or higher is defined as glomerular filtration <60mL/min/1.73m2; ^§^ within a period of 30 days after diagnosis of sepsis, the occurrence of death, or discharge from ICU, whichever came first.

**Table 2:** Cox regression analysis assessing the association of variables with 90-day mortality in patients with sepsis (n=282).

	Univariate		Multivariate			
			Initial		Restricted	
Covariate	Hazard Ratio (95% CI)	*p*-Value	Hazard Ratio (95% CI)	*p*-Value	Hazard Ratio (95% CI)	*p*-Value
**Aquaporin 5 –1364A/C genotype**						
- AA	1	-	1	-	1	-
- AC/CC	0.60 (0.42 to 0.86)	0.005	0.61 (0.42 to 0.88)	0.008	0.60 (0.42 to 0.86)	0.006
**Sex**						
- Women	1	-	1	-		
- Men	1.09 (0.77 to 1.53)	0.628	1.40 (0.98 to 2.00)	0.067		
**Age** [years]						
- <60	1	-	1	-		
- ≥60	1.09 (0.78 to 1.52)	0.610	1.21 (0.82 to 1.81)	0.335		
**CKD of stage 3 or higher** ^§^						
- No	1	-	1	-		
- Yes	1.47 (0.93 to 2.32)	0.260	1.01 (0.58 to 1.76)	0.971		
Vasopressor support*						
- No	1	-	1	-	1	-
- Yes	4.64 (2.05 to 9.9)	<0.001	2.57 (1.09 to 6.07)	0.031	3.01 (1.30 to 7.00)	0.010
Mechanical Ventilation*						
- No	1	-	1	-	1	-
- Yes	3.75 (2.02 to 6.94)	<0.001	2.50 (1.32 to 4.71)	0.005	2.57 (1.37 to 4.83)	0.003
**Net fluid balance** ^$^						
- +1.0L to −1.0L	1	-	1	-	1	-
- < −1.0L	0.41 (0.26 to 0.66)	<0.001	0.45 (0.28 to 0.73)	0.001	0.42 (0.26 to 0.68)	<0.001
- > +1.0L	1.64 (1.13 to 2.38)	0.009	1.81 (1.24 to 2.65)	0.002	1.74 (1.20 to 2.53)	0.004
**Serum lactate concentration** [mg/dL]*						
- <2.0	1	-	1	-	1	-
- ≥2.0	2.40 (1.68 to 3.44)	<0.001	2.13 (1.45 to 3.13)	<0.001	2.29 (1.59 to 3.31)	<0.001
**Total bilirubin concentration** [mg/dL]*						
- <2.0	1	-	1	-		
- ≥2.0	1.64 (1.16 to 2.31)	0.005	1.30 (0.89 to 1.90)	0.170		
**Acute Kidney Injury** ^$^						
- No AKI	1	-	1	-		
- AKI 1+2	1.99 (1.05 to 3.79)	0.036	1.26 (0.64 to 2.48)	0.496		
- AKI 3	3.13 (1.71 to 5.73)	<0.001	1.69 (0.87 to 3.28)	0.122		
**Procalcitonin con**. [pg/mL]*	1,00 (0.99 to 1.00)	0.763	1.00 (0.99 to 1.00)	0.875		

HR: Hazard ratio point estimates, 95% CI, and *p*-values (two-sided) are reported; CKD: Chronic kidney disease; Omnibus test of model coefficients: Chi-square = 93.7, *p* < 0.001 and Homer–Lemeshow statistics for the restricted multivariable approach were as follows: κ2 = 2.675; *p* = 0.953. ^§^ Status prior to sepsis; * within 24 h after diagnosis of sepsis (day1); ^$^ Within a period of 30 days after diagnosis of sepsis, the occurrence of death, or discharge from ICU, whichever came first.

## Supplementary Materials

The following are available online at https://www.mdpi.com/2073-4409/9/4/904/s1, Table S1: Baseline characteristics of septic patients with and without acute renal failure (n = 282), Table S2: Baseline characteristics of septic patients stratified for 90-day survival and non-survival (n = 282).
