# Supplementary material for: Major Adverse Kidney Events Are Associated with the Aquaporin 5 -1364A/C Promoter Polymorphism in Sepsis: A Prospective Validation Study
Source: Cells. 2020 Apr 7;9(4):904. doi: 10.3390/cells9040904 (PMC7226758; doi:10.3390/cells9040904)
Supplement: Supplementary file 1 [file cells-09-00904-s001.zip › TableS1.pdf]

Table S1: Baseline characteristics of septic patients with and without acute renal failure (n=282)

Characteristic	AKI (n =235)		No AKI (n = 47)		p-value
Age [years]	58.2	(±14.9)	50.3	(±15.3)	0.001
Sex, male [n]	151	(64.3%)	23	(48.9%)	0.045
Body mass index [kg/m2]	27.1	(±5.6)	27.0	(±6.1)	0.890
Ethnicity [n]					0.525
- Caucasian	225	(95.7%)	44	(93.6%)	
- Other	10	(4.3%)	3	(6.4%)	
AQP5 genotype [n]					0.001
- AA	150	(63.8%)	17	(36.2%)	
- AC	76	(32.4%)	24	(51.1%)	
- CC	9	(3.8%)	6	(12.7%)	
Medical history [n]					
- Cardiovascular disease	134	(57.0%)	22	(46.8%)	0.197
- Pulmonary disease	57	(24.3%)	14	(29.8%)	0.425
- Diabetes mellitus	44	(18.7%)	7	(14.9%)	0.678
- Gastrointestinal disease	32	(13.6%)	9	(19.1%)	0.457
- History of malignant disease	20	(8.5%)	5	(10.6%)	0.851
- CKD of stage 3 or higher$ [n]	34	(14.5%)	0	n/a	0.002
SAPS II score	42.6	(±18.2)	37.7	(±17.3)	0.092
SOFA score	12.6	(±4.2)	9.1	(±4.2)	<0.001
Septic Shock [n]	57	(24.4%)	4	(8.5%)	0.019
Vasopressor support [n]	213	(90.6%)	33	(70.2%)	<0.001
Mechanical ventilation [n]	196	(83.4%)	32	(68.1%)	0.015
Net fluid balance [L]	0.0	(-1.6 to 1.5)	-0.4	(-2.0 to 1.0)	0.123
Procalcitonin concentration [pg/mL]	5.8	[1.8-16.5]	1.5	[0.6-3.0]	<0.001
C-reactive protein concentration [mg/dl]	13.0	[7.2-21.3]	16.6	[10.2-25.2]	0.216
Leukocyte concentration [nl-1]	13.6	[9.0-19.6]	13.3	[8.9-21.6]	0.814
Hemoglobin [g/dL]	9.4	[8.9-10.5]	9.6	[8.6-10.7]	0.821
Total bilirubin concentration [mg/dL]	1.1	[0.5-2.5]	0.8	[0.4-1.5]	0.048
Serum-lactate concentration [mg/dL]	1.4	[0.8-1.9]	1.0	[0.5-1.5]12	0.011
Etiology of infection [n]					0.748
- Pneumonia	86	(36.6%)	20	(42.6%)	
- Urinary tract infection	63	(26.8%)	8	(17.0%)	
- Abdominal infection	35	(14.9%)	9	(19.1%)	
- Skin or muscle infection	11	(4.7%)	3	(6.4%)	
- Bloodstream infection	10	(4.2%)	2	(4.3%)	
- Other / unknown origin	30	(12.8%)	5	(10.6%)	
Blood cultures [n]					0.622
- Gram-positive isolates	64	(27.2%)	15	(31.9%)	
- Gram-negative isolates	65	(27.7%)	11	(23.4%)	
- Fungal isolates	10	(4.2%)	1	(2.1%)	
- Mixed isolates	53	(22.6%)	8	(17.0%)	
- Negative blood cultures	43	(18.3%)	12	(25.6%)	

The data are presented as n (%), mean (± SD), or median (25th - 75th percentile). SOFA score: Sepsis- related Organ Failure Assessment score; SAPS II score; Simplified Acute Physiology score. The following missing data were excluded from the analysis: 4 case missing for body mass index; 5 cases were missing for SAPS II score; 8 cases missing for procalcitonin concentration; 16 cases missing for C-reactive protein concentration; 7 cases missing for leukocyte concentration; 17 cases missing for blood urea nitrogen. $ Chronic kidney disease of stage 3 or higher is defined as glomerular filtration <60mL/min/1.73m2.
