# Supplementary material for: Major Adverse Kidney Events Are Associated with the Aquaporin 5 -1364A/C Promoter Polymorphism in Sepsis: A Prospective Validation Study
Source: Cells. 2020 Apr 7;9(4):904. doi: 10.3390/cells9040904 (PMC7226758; doi:10.3390/cells9040904)
Supplement: Supplementary file 1 [file cells-09-00904-s001.zip › TableS2.pdf]

Table S2: Baseline characteristics of septic patients stratified for 90-day survival and non-survival (n=282)

**Characteristic**	**Survivors** (n = 142)		**Non-Survivors** (n = 140)		**p-value**
Age [years]	58.1	(±15.2)	55.7	(±15.3)	0.189
Sex, male [n]	88	(62.0%)	86	(61.4%)	0.925
Body mass index [kg/m2]	27.4	(±5.8)	26.8	(±5.5)	0.367
Ethnicity [n]					0.167
- Caucasian	138	(97.2%)	131	(93.6%)	
- Other	4	(2.8%)	9	(6.4%)	
AQP5 genotype [n]					0.015
- AA	73	(51.4%)	94	(67.1%)	
- AC	58	(40.8%)	42	(30.0%)	
- CC	11	(7.8%)	4	(2.9%)	
Medical history [n]					
- Cardiovascular disease	72	(50.1%)	84	(60.0%)	0.116
- Pulmonary disease	31	(21.8%)	40	(28.6%)	0.192
- Diabetes mellitus	23	(16.2%)	28	(20.0%)	0.406
- Gastrointestinal disease	23	(16.2%)	18	(12.9%)	0.426
- History of malignant disease	12	(8.5%)	13	(9.3%)	0.805
Renal conditions					
- CKD of stage 3 or higher$ [n]	12	(8.5%)	22	(15.7%)	0.061
SAPS II score	39.0	(±18.8)	44.7	(±17.1)	0.009
SOFA score	11.0	(±4.5)	13.1	(±4.0)	<0.001
Septic Shock [n]	17	(12.0%)	44	(31.4%)	<0.001
Vasopressor support [n]	112	(78.9%)	134	(95.7%)	<0.001
Mechanical ventilation [n]	99	(69.7%)	129	(92.1%)	<0.001
Net fluid balance [L]	-1.2	(-2.1 to 0.0)	0.8	(-0.1 to 1.8)	<0.001
Procalcitonin concentration [pg/mL]	3.2	[1.3-12.4]	5.7	[1.6-16.0]	0.056
C-reactive protein concentration [mg/dl]	12.7	[7.8-24.4]	14.6	[7.3-20.8]	0.910
Leukocyte concentration [nl^{-1}]	12.7	[9.4-17.9]	15.2	[8.3-21.1]	0.318
Creatinine concentration [mg/mL]	1.27	[0.74-1.99]	1.53	[0.96-2.22]	0.028
Blood urea nitrogen [mg/dL]	17.1	[11.3-25.3]	19.0	[12.4-29.2]	0.202
Hemoglobin [g/dL]	9.4	[8.9-10.5]	9.5	[8.7-10.6]	0.926
Total bilirubin concentration [mg/dL]	0.9	[0.4-1.6]	1.3	[0.5-3.1]	0.001
Serum-lactate concentration [mg/dL]	1.0	[0.5-1.5]	1.5	[0.9-2.7]	<0.001
Renal-Replacement Therapy [n]	45	(31.7%)	81	(57.9%)	<0.001
AKI stage [n]					0.001
- No AKI	35	(24.7%)	12	(8.6%)	
- AKI 1	31	(21.8%)	22	(15.7%)	
- AKI 2	21	(14.8%)	19	(13.6%)	
- AKI 3	55	(38.7%)	87	(62.1%)	

The data are presented as n (%), mean (± SD), or median (25_{th}-75_{th} percentile). SOFA score: Sepsis- related Organ Failure Assessment score; SAPS II score; Simplified Acute Physiology score. The following missing data were excluded from the analysis: 4 case missing for body mass index; 5 cases were missing for SAPS II score; 8 cases missing for procalcitonin concentration; 16 cases missing for C-reactive protein concentration; 7 cases missing for leukocyte concentration; 17 cases missing for blood urea nitrogen. $ Chronic kidney disease of stage 3 or higher is defined as glomerular filtration <60mL/min/1.73m_2.
